# Carotid endarterectomy with active extracorporeal perfusion for bilateral ocular ischemic syndrome in a patient with a preoperatively defined critical single-vessel cerebral supply on four-dimensional flow magnetic resonance imaging

**DOI:** 10.1016/j.jvscit.2026.102180

**Published:** 2026-02-12

**Authors:** Johan Millinger, Isabella M. Björkman-Burtscher, Anna Corderfeldt Keiller, Elias Johansson, Kerstin Lagerstrand, Joakim Nordanstig

**Affiliations:** a Department of Molecular and Clinical Medicine, Institute of Medicine, University of Gothenburg, Gothenburg, Sweden; b Department of Vascular Surgery, Sahlgrenska University Hospital, Gothenburg, Sweden; c Department of Radiology, Institute of Clinical Sciences, Sahlgrenska Academy, University of Gothenburg, Gothenburg, Sweden; d Department of Radiology, Sahlgrenska University Hospital, Gothenburg, Sweden; e Institute of Clinical Sciences, Sahlgrenska Academy, University of Gothenburg, Gothenburg, Sweden; f Department of Perfusion, Sahlgrenska University Hospital, Gothenburg, Sweden; g Department of Neuroscience and Physiology, Gothenburg University, Gothenburg, Sweden; h Department of Biomedical Engineering and Medical Physics, Sahlgrenska University Hospital, Gothenburg, Sweden

**Keywords:** Carotid artery stenosis, Perfusion/methods, Perfusion MRI, Vascular surgical procedures

## Abstract

Ocular ischemic syndrome is a rare manifestation of severe carotid artery occlusive disease with risk of vision loss. We report a patient with bilateral ocular ischemic syndrome secondary to advanced atherosclerosis, including brachiocephalic and subclavian artery occlusions and high-grade bilateral carotid stenosis. Four-dimensional flow magnetic resonance imaging demonstrated near-exclusive cerebral perfusion derived from the left carotid system. Carotid endarterectomy was performed using extracorporeal veno-arterial antegrade selective cerebral perfusion for neuroprotection, as standard shunting was deemed inadequate. This case highlights the utility of four-dimensional flow magnetic resonance imaging in identifying hypoperfusion risk and the utility of extracorporeal cerebral perfusion in complex carotid revascularization.

Ocular ischemic syndrome (OIS) is a rare but potentially vision-threatening condition caused by severe steno-occlusive carotid disease leading to chronic retinal hypoperfusion. Common clinical features include progressive vision loss, transient visual obscuration, or ischemic ocular pain.^1^^,^^2^

Beyond ophthalmologic manifestations, OIS carries significant systemic implications as it involves carotid stenosis, a known risk factor for stroke and other atherosclerotic diseases.^3^ The estimated incidence is 7.5 cases per million annually, and bilateral involvement has been reported in up to 20% of cases.^2^ Severity depends on stenosis degree, adequacy of collateral circulation, chronicity of the carotid disease, and comorbid vascular disease.^2^

Carotid endarterectomy (CEA) remains the standard of care for symptomatic high-grade stenosis.^3^^,^^4^ However, cerebral protection during CEA remains debated. Shunt use varies widely, predicting which patients need shunting before surgery is challenging, and the global flow capacity of commonly used shunts may sometimes also be inadequate.^5^

We report on a patient with advanced atherosclerotic disease in the supra-aortic vessels who underwent open CEA. On preoperative computed tomography angiography (CTA), a dependence on the left carotid system was suspected, including reliance on flow in the external carotid artery (ECA). This suspicion was confirmed on a preoperative four-dimensional (4D) flow magnetic resonance imaging (MRI) assessment of cerebral hemodynamics. Based on these findings, extracorporeal circulation (ECC) with veno-arterial antegrade selective cerebral perfusion—from the femoral vein to both the internal (ICA) and ECA on the left side—was employed intraoperatively to ensure adequate cerebral perfusion. The patient has consented to the publication of this case report.

## Case report

A 78-year-old woman (166 cm, 54 kg) with a history with prior percutaneous coronary intervention for non-ST-segment elevation myocardial infarction (2018), chronic heart failure, hypertension, dyslipidemia, and ischemic stroke (2001), with transient left-sided weakness. Related to this, she had previously undergone uneventful right-sided CEA and was maintained on clopidogrel monotherapy. Laboratory findings revealed thrombocytopenia in the context of low-grade myelodysplastic syndrome.

In 2022, the patient developed progressive right eye visual impairment, followed later by deterioration also in the left eye. Bilateral cataract surgery was performed without improvement. Neuro-ophthalmologic evaluation established the diagnosis of bilateral OIS, now with complete visual loss in the right eye and partial loss in the left. The right eye was deemed beyond rescue, but the vision in the left eye was possibly salvageable. She was referred for multidisciplinary assessment and consideration of carotid revascularization following duplex ultrasonography and CTA. The patient appeared vital and cognitively intact during assessment.

### Investigations

**Fig 1:**
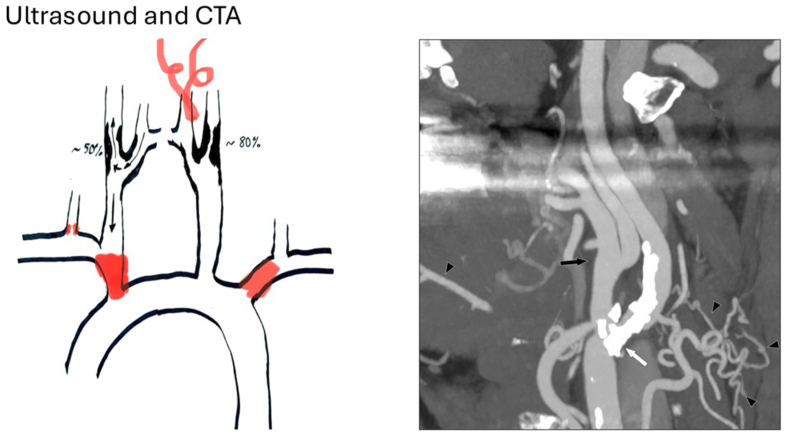
(Left) Schematic drawing of arterial occlusions, stenoses, and direction of flow in the carotid circulation. (Right) Computed tomography angiography (*CTA*), coronal view. Heavily calcified stenosis in proximal internal carotid artery (*white arrow*). Notice the unusually large external carotid artery (ECA) (*black arrow*) and several prominent branches (*black arrowheads*) that seemed to feed collaterals, including anastomoses to the vertebral arteries.

**Fig 2:**
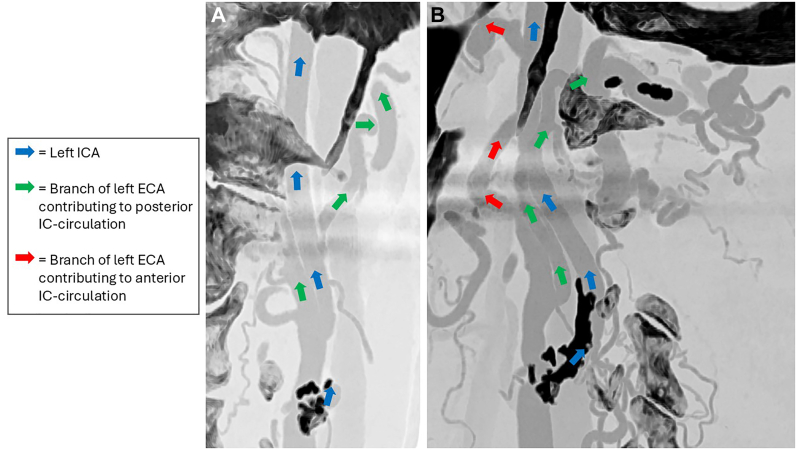
Computed tomography angiography (CTA) showcasing extensive collaterals from the left external carotid artery (*ECA*). **(A)** Frontal view. **(B)** Sagittal view. *IC*, Intracranial, *ICA*, internal carotid artery.

Duplex ultrasonography revealed high-grade (70%-99%) left ICA stenosis and a moderate (50%-69%) right ICA restenosis, with reversed flow in the right ECA and common carotid artery (CCA). CTA showed innominate artery and left subclavian artery occlusions, severe stenosis of the right vertebral artery (Fig 1), and extensive collateral circulation from the left ECA to the intracranial circulation (Fig 2). Blood pressure measurements demonstrated marked discrepancies: systolic blood pressure was 50 mmHg (right arm), 60 mmHg (left arm), 200 mmHg (right leg), and 190 mmHg (left leg).

**Fig 3:**
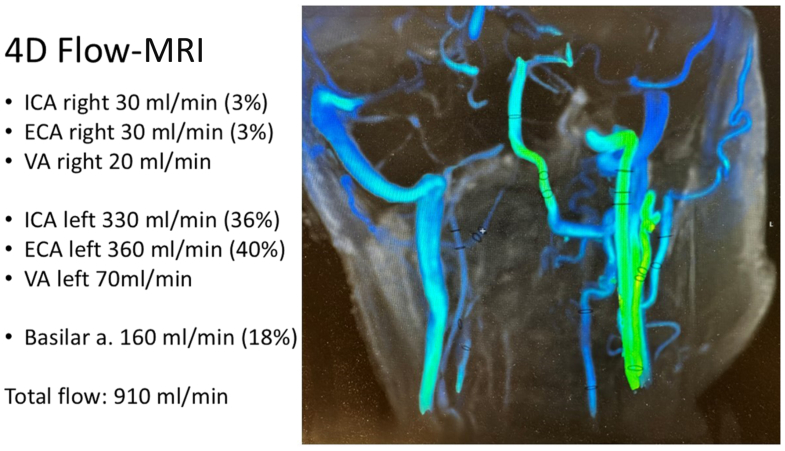
Four-dimensional (4D) flow magnetic resonance imaging (MRI) was performed using a commercial gradient echo cardio gated sequence (TR/TE = 4.7/2.6 ms; field-of-view = 240 × 180 mm^2^; voxel size = isotropic 1.2 mm^3^; temporal resolution 83 ms; 228 slices; BW/pixel = 625 Hz; total acquisition time = 7:57 minutes). A 3 T clinical scanner (Premier, GE HealthCare) was used. To enhance the visibility of the vessels, Gadobuturol 1.0 mmol/mL (Bayer AG) per 1 mmol/kg bodyweight was continuously administered during 4D flow MRI. The postprocessing of the 4D flow dataset was mainly performed using Cardio AI MR (Arterys). Four-D Flow MRI showed predicted volume-flow in internal carotid artery (*ICA*), external carotid artery (*ECA*), and vertebral artery (*VA*) on both left and right sides and predicted volume-flow in the Basilar artery (*Basilar a*).

Both the stroke neurologist and the neuro-ophthalmologist recommended left-sided carotid revascularization, as loss of vision on that eye would result in complete blindness. The multidisciplinary team identified left ECA flow as possibly essential for maintaining cerebral and ocular perfusion, by indirect signs on routine imaging. Given the possible technical implications, a 4D flow MRI was conducted. It demonstrated total head blood flow of 910 mL/min, with 76% derived from the left CCA—40% via the ECA and 36% via the ICA (Fig 3). A conventional cerebral angiography was not performed.

Subclavian artery recanalization was considered but rejected, as it would not improve left carotid flow and carries an inherent stroke risk. Given the high risk associated with compromising ECA flow, carotid artery stenting was excluded as a treatment option, favoring open surgical revascularization. However, conventional clamping of the left carotid artery would then be unsafe. Conventional carotid shunting to the ICA might not be sufficient, regarding volume blood flow, in this case were major collaterals from the ECA to the posterior intracranial circulation would not be provided for in a safe manner. Blood supply to the posterior intracranial circulation is more difficult to monitor during surgery. Conventional carotid shunting also carries known flow limitations^3^, ^4^, ^5^ that might have severe consequences in a case where most of the brain derives its blood supply from a single carotid artery. Given the prophylactic nature of the operation and to mitigate perioperative risk, the cardiothoracic perfusionist team was consulted, and a strategy incorporating extracorporeal active selective cerebral perfusion was devised.

### Surgical procedure

The operation was performed under general anesthesia utilizing intraoperative cerebral oximetry (INVOS) as neuromonitoring. Venous access was obtained via the left femoral vein, and arterial pressure monitoring was established via the right femoral artery. Wide exposure of the carotid bifurcation and ECA was achieved, and intraoperative flow measurements confirmed preoperative MRI findings, (±10 mL/min).

**Fig 4:**
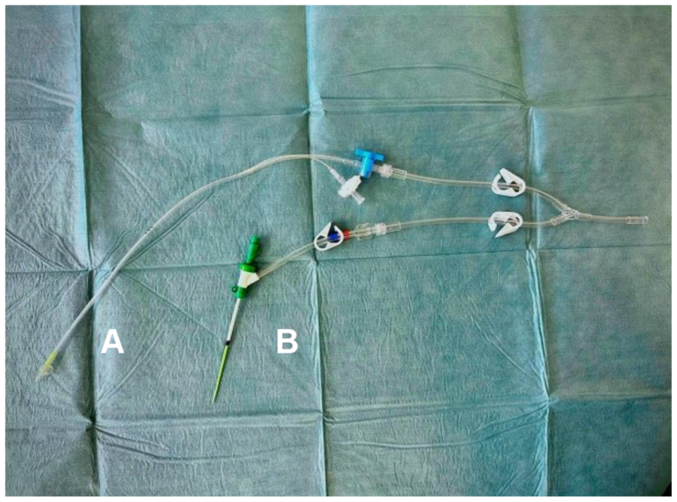
The distal components of the extracorporeal circulation (ECC)-circuit. **(A)** A 9 Fr Pruitt irrigation occlusion catheter (LeMaitre) inserted into the left internal carotid artery (ICA). **(B)** A 6 Fr Prelude short-sheath introducer (Merit Medical) inserted into the left external carotid artery (ECA).

After systemic heparinization (10,000 IU), a 6 Fr Prelude short-sheath introducer (Merit Medical) was inserted into the proximal ECA via Seldinger technique. This introducer was chosen for its short length and favorable volume-flow characteristics.^6^ The extracorporeal circuit, a S5 LivaNova (LivaNova) with roller pump, a pediatric Pixie oxygenator with 2 L reservoir (Medtronic) connected with 1/4-inch tubing was primed with 250 mL Plasma-Lyte, 250 mL bank blood, and 100 mL Tribonate. Venous drainage was achieved via a 15 Fr malleable femoral vein catheter (Medtronic). An additional 2500 IU heparin maintained an activated clotting time of ∼240 seconds. ECC commenced with 350 mL/min flow to the ECA, with supplemental oxygenation and circuit cooling (33°C) for added cerebral protection. The flow rate was chosen based on results from the 4D flow MRI and the intraoperative flow measurements. The ECC was started prior to clamping of the ECA proximally to the access, minimizing ischemic insult. Temporary clamping of the CCA caused a significant INVOS decline (>20%), necessitating additional shunting into the ICA. Following a longitudinal incision in the CCA extending into the ICA above the stenosis, a 9 Fr Pruitt irrigation occlusion catheter (LeMaitre) was inserted into the ICA and connected to the ECC circuit, increasing total flow to 500 mL/min (Fig 4). Subsequently, the INVOS values returned to pre-procedural values and remained stable throughout.

A standard CEA with patch angioplasty was performed. Post-unclamping, flow measurements revealed 750 mL/min in the ICA and 720 mL/min in the ECA. Hemostasis was secured, and the procedure was completed without intraoperative complications. The postoperative course was uneventful. Antihypertensive therapy required minor adjustments. Length of stay in hospital was 8 days. At 1-month follow-up, the patient remained clinically stable, and at 3 months, neuro-ophthalmologic assessment confirmed preserved visual acuity in the left eye.

## Discussion

This case highlights the feasibility and the potential role of 4D flow MRI and ECC-assisted selective cerebral perfusion in complex carotid revascularization.

Predicting the need for intraoperative shunting during carotid surgery remains challenging,^5^ as does estimating the cerebral blood flow required for adequate perfusion. One of the most used carotid shunts, the Pruit-Inahara shunt (LeMaitre), referring to the manufacturer’s instructions for use, provides a flow capacity of 180 to 220 mL/min at a specified blood pressure of 120/80 mmHg. The human brain typically requires 15% of the cardiac output, which corresponds to approximately 750 mL/min.^7^^,^^8^ In many cases, collateral circulation via the contralateral carotid artery and vertebral arteries is sufficient to maintain cerebral perfusion without shunt need. In selected patients, however, shunting becomes necessary.^3^^,^^4^

Four-D flow MRI may serve as a valuable adjunct in preoperative assessment by identifying patients at risk of hypoperfusion and predicting when conventional shunts may prove inadequate.^5^^,^^6^ In this case, intraoperative flow measurements closely mirrored preoperative 4D flow MRI findings, confirming near-exclusive cerebral perfusion via the left carotid system. Selective antegrade cerebral perfusion using an ECC circuit is an established technique in cardiac surgery.^9^, ^10^, ^11^ However, this approach is rarely applied in vascular surgery but was successfully used in our patient with single-vessel cerebral perfusion who required open CEA. Advantages of the technique include the ability to measure and directly control blood flow, while simultaneously providing selective oxygen enrichment and hypothermic protection of the cerebral circulation.

## Conclusions

Carotid revascularization in patients with single-vessel cerebral supply poses major challenges, as conventional shunting may not ensure adequate perfusion. Preoperative 4D flow MRI can help assess cerebral flow requirements and guide individualized strategies. ECC-assisted selective antegrade cerebral perfusion provided safe and controlled revascularization in this high-risk case and represents a viable alternative in selected patients where standard methods are insufficient.

## Funding

None.

## Disclosures

None.
